# Varieties of Young Children’s Prosocial Behavior in Zambia: The Role of Cognitive Ability, Wealth, and Inequality Beliefs

**DOI:** 10.3389/fpsyg.2018.02209

**Published:** 2018-11-16

**Authors:** Nadia Chernyak, Teresa Harvey, Amanda R. Tarullo, Peter C. Rockers, Peter R. Blake

**Affiliations:** ^1^Department of Psychology, Boston College, Newton, MA, United States; ^2^Department of Cognitive Sciences, University of California, Irvine, Irvine, CA, United States; ^3^Department of Psychological and Brain Sciences, Boston University, Boston, MA, United States; ^4^Department of Global Health, Boston University School of Public Health, Boston, MA, United States

**Keywords:** Zambia, prosocial behavior, inequality, cross-cultural developmental psychology, preschoolers

## Abstract

By the 3rd year of life, young children engage in a variety of prosocial behaviors, including helping others attain their goals (instrumental helping), responding to others’ emotional needs (comforting), and sharing resources (costly giving). Recent work suggests that these behaviors emerge early, during the first 2 years of life (Svetlova et al., 2010; Thompson and Newton, 2012; Dunfield and Kuhlmeier, 2013). To date, however, work investigating early varieties of prosocial behavior has largely focused on Western samples and has not assessed the impact of poverty and inequality. In this work, we investigate prosocial behavior in 3-year-olds in Zambia, a lower-middle income country with high wealth inequality. Experiments were integrated into a larger public health study along with both objective and subjective (parent) measures of wealth and inequality. Three-hundred-seventy-seven children (*Mean age* = 36.77 months; *SD* = 2.26 months) were presented with an instrumental helping task, comforting task, and two steps of a giving task – one with higher cost (children could give away their only resource) and one with lower cost (children had three resources to give). As predicted, rates of prosociality varied hierarchically by the cost of the action: instrumental helping was the most common followed by comforting, lower cost giving, and higher cost giving. All prosocial behaviors were significantly correlated with one another (with the exception of high cost giving), and with general cognitive ability. Objective family wealth did not predict any of the child’s prosocial behaviors. However, subjective beliefs showed that mothers who believed that they had more than others in their village had children who were more likely to engage in instrumental helping, and mothers who believed that village inequality was a problem had children who were more likely to engage in low cost giving. Low cost giving was also more likely for children whose parents reported reading storybooks to them. This suggests that costly giving in the context of pretend play may relate to children’s experience with using stories as representations of real life events. The results suggest both cultural differences and universalities in the development of prosociality and point to environmental factors that influence prosociality.

## Introduction

By the 3rd year of life, young children show a variety of prosocial behaviors, including helping others attain their goals (instrumental helping), responding to others’ emotional needs (comforting), and sharing resources (costly giving). Recent work generally finds that instrumental helping and comforting behaviors, which are lower in cost to self (i.e., effort), develop earlier than costly giving. Giving is considered more costly because one must sacrifice material resources to benefit others. These findings suggest that the development of humans’ prosocial behavior proceeds in accordance with how costly it is to oneself (Warneken and Tomasello, 2009; Sommerville et al., 2018). To date, however, work investigating early varieties of prosocial behavior has largely focused on Western and relatively wealthy samples. As such, this work leaves open important research questions that comprised the aim of the current work: (1) to what extent is the development of these three forms of prosocial behavior similar in diverse societies and (2) how do demographic variables, such as wealth, affect children’s prosocial behavior within a society. A third question concerns the role that parents’ subjective beliefs about their economic status plays in shaping children’s prosociality. We investigate these questions in a large-scale sample of Zambian children in order to test these predictions in a sample markedly different from the Western societies in which these questions have previously been studied, as well as to study the impact of local and global inequality on prosocial behavior.

Using both evolutionary and developmental evidence, a hierarchy of prosocial behaviors has been proposed based on the cost of different actions, with helping as relatively low cost and giving away resources as the highest cost (Warneken and Tomasello, 2009). The cost of an action even appears to moderate behaviors within the distinct subtypes of prosociality. For example, 18- and 30-month-olds are more likely to help others when they do not have to give up their own property to do so (Svetlova et al., 2010). For giving, preschoolers give away more of a resource that they value less compared to a resource they value more (Blake and Rand, 2010). Combined these studies suggest a hierarchy of prosociality based on cost to the actor that is evident early in development.

While most studies on children’s prosociality have been conducted with Western samples, the limited cross-cultural evidence supports the idea that prosocial behaviors comprise separate subtypes, and that cultural differences in rates of prosocial behavior emerge as the cost of the behavior increases. For example, experiments have found that, similar to Western samples, low cost forms of helping are apparent by 18 months of age in rural communities in India, Peru and Brazil as well as in Western urban communities (Callaghan et al., 2011; Moritz et al., 2016), though notably, rates of prosocial behaviors differed among these samples (Callaghan et al., 2011). Low cost forms of giving (a choice of 1 for self, 0 for a peer vs. 1 for each) have also been found for children in hunter-gatherer, pastoralist and horticultural societies (House et al., 2013). By contrast, high cost forms of giving (a choice of 2 for self, 0 for a peer vs. 1 for each) varied across the same six societies (House et al., 2013). Children in seven diverse societies have also been found to engage in costly enforcement of equality when they face a disadvantage relative to a peer, but cultural variation in fairness enforcement appears when children face an advantage over a peer, a relatively higher cost (Blake et al., 2015a; Corbit et al., 2017). Cultural similarities have also been found in children’s willingness to imitate low cost forms of giving, but differences emerge when the costs increase (Blake et al., 2016). Combined these results suggest a universal willingness to engage in prosocial behavior in early childhood that varies across societies as the cost of the prosocial behavior increases. However, a complete test of the hierarchy of costs model for all three subtypes of prosociality has not been conducted outside of Western societies.

Adding new societies for cross-cultural comparisons of development remains an important goal of psychological research (Correa-Chavez and Rogoff, 2005; Nielsen et al., 2017), but variation within a society is also important for examining the effects of environmental variables on children’s behavior (e.g., see Alcalá et al., 2014). For example, prior cultural work has observed that children’s prosocial behavior is deeply affected by their abilities to observe, participate, and learn from the chores and responsibilities that affect the adults around them (Silva et al., 2010). In Southern Zambia, where this project took place, young children are traditionally expected to help (e.g., gather and carry firewood) from as soon as they can walk and are encouraged to share by adults (Colson, 1967). By 4–5 years of age, children are expected to begin household duties based on gender: boys begin herding livestock and girls help with childcare and planting. Despite these traditional values of work and helping, children in contemporary Zambian society also face varying degrees of resource inequality and malnutrition with high rates of stunted growth in the country as a whole (Rockers et al., 2018). These local and global influences may affect the rates of prosociality, either by increasing rates of instrumental helping relative to Western samples, or by decreasing rates of resource sharing, due to exposure to resource inequality and scarcity.

For prosocial behavior in particular, socioeconomic status (SES) has been proposed as an important within-culture predictor for both adults and children, though prior work has found opposing effects of SES on prosociality. For example, some studies have found that adults with higher SES are less prosocial (Frank, 1999; James and Sharpe, 2007; Piff et al., 2010), but a large scale cross-national analysis found that wealthier adults are more prosocial compared to low SES individuals (Korndörfer et al., 2015). The same inconsistency has been found in developmental samples as well: children of wealthy families have been found both to give more (Benenson et al., 2007; Safra et al., 2016) and less (Miller et al., 2015) compared to low SES children. Moreover, one cross-cultural study found that both the poorest children (street children in Recife, Brazil) and the wealthiest children (from private day care in the United States) gave the fewest resources to a recipient in a Dictator Game (Rochat et al., 2009).

One potential moderating factor that may explain these conflicting findings is income inequality, although limited work has examined these effects on children. Wealthy adults in areas of high inequality tend to be less prosocial compared to wealthy adults in low inequality regions (Côté et al., 2015). Reviews of research on wealth and inequality have also found that subjective perceptions of social status can impact a range of outcomes (Kraus et al., 2011). In addition, work on so called relative deprivation suggests that perceptions of oneself as being low social status relative to others (subjective social status; SSS) can have negative effects on behavior, and cognitive functioning in particular (Heberle and Carter, 2015). Perceptions of SSS are likely to be formed by adults, whose beliefs may influence children’s behavior. Although speculative, we examined this possibility for children’s prosocial behavior in the current study. In particular, we investigated the possibility that subjective beliefs about inequality might predict children’s sharing behavior, for either low or high cost giving. Because no work to our knowledge has investigated or found relationships between inequality and other forms of prosocial behavior, we did not expect inequality beliefs to predict instrumental helping or comforting.

In summary, research on children’s prosociality has identified three primary forms that emerge before or by 3 years of age: instrumental helping (i.e., helping others achieve a goal; Warneken and Tomasello, 2006), comforting (i.e., sympathizing and offering help to those in distress; Svetlova et al., 2010; Dunfield, 2014), and costly resource sharing (Blake and Rand, 2010; Chernyak and Kushnir, 2013; Chernyak et al., 2017, 2018). Although these behaviors appear in a range of societies, all three forms have not been tested in single non-Western society. Moreover, these behaviors may vary based on the degree of wealth and inequality experienced by a given family. In the current study, we conducted a test of the hierarchy of costs model of prosocial behavior in Zambia, a lower-middle income country with high inequality of wealth. Prosocial experiments and parent questionnaires were added to the second wave of data collection of a public health intervention. We thus obtained both objective and subjective measures of family wealth, parent beliefs about status and inequality, and measures of parenting practices.

### Research Context

As a country, Zambia is marked by both high poverty and high wealth inequality. According to the World Bank indicators database, approximately 60% of the population lives below the poverty line and income inequality is among the highest in the world (Gini coefficient = 0.57 in 2015; compared with United States Gini coefficient = 0.41 and Canada Gini coefficient = 0.34)^1^. In the rural areas where the study was conducted, the primary occupation is farming. Households often grow their own food on small plots of land and sell the excess at roadside markets. Families typically have limited childcare and education and public health research has found high rates of childhood stunted growth (Rockers et al., 2018).

The current study added measures to a health intervention targeting households with children between 6 and 12 months of age at baseline. The intervention occurred over 2 years with the treatment group attending bi-weekly parenting groups to learn about cognitive enrichment and play activities for their children, nutrition and self-care. The control group received no intervention. The original study was implemented as a cluster-randomized controlled trial in the Southern Province of Zambia, specifically the districts of Choma and Pemba. The clusters were 30 health zones and were randomized to treatment or control prior to enrollment. Villages within each zone were randomly selected and within those villages all eligible households were asked to participate. Participation was voluntary and all caregivers were provided written informed consent prior to study initiation. The study was approved by the Institutional Review Board at Boston University (protocol number H-32726) and by the ethics board at ERES Converge in Zambia (protocol number 2013-Dec-010) prior to the enrollment of participants. At the end of the 2-year period, study participants were re-consented for the final wave of data collection which included the measures added to the procedure for the current study.

At the beginning of the health intervention there were 268 mother-child dyads in the intervention group and 258 dyads in the control group. By the final wave of data collection there were 195 dyads in the intervention group (73%) and 182 dyads in the control group (71%). The total sample reported here thus consists of 377 mother-child pairs.

## Materials and Methods

### Participants

Participants were 377 toddlers (190 males, 187 females; *Mean age* = 36.77 months; *Range* = 31.77 – 41.57 months) recruited through a larger public health study on maternal and child health outcomes. All mothers were provided with questionnaires about their own and their child’s physical and emotional well-being, beliefs about parenting, and beliefs about inequality and interpersonal trust. Children were administered standardized measures of health, including the Bayley Scales of Infant and Toddler Development, Third Edition (BSID-III) as well as measured for their height, weight, and mid-upper arm circumference.

### Procedure

In addition to questionnaires and assessments aimed at investigating maternal and child health (not discussed or analyzed here), our group added the following structured tasks to the assessments which serve as the focus of this paper. Tasks were adapted from prior work aimed at studying prosocial behavior within this age group, and designed by the authors in consultation with local researchers who helped to make critical design modifications in order to make the tasks ecologically appropriate for the sampled population (e.g., using toys instead of stickers as the resource).

#### Instrumental Helping Task

In this task, adapted from Warneken and Tomasello (2006), the experimenter appeared to drop a bunch of sticks in front of the child seemingly by accident. The experimenter then expressed 4 cues in successive order to solicit the child’s help. Each cue was followed by a 10-s pause in order to allow the child an opportunity to respond. During the first cue, the experimenter simply stared at the sticks and exclaimed “Oops.” During subsequent cues, the experimenter alternated between looking at the sticks and the child and said “I dropped my sticks” (second cue), “I dropped my sticks, I need them back” (third cue), and “Can you help me get my sticks?” (fourth cue; with palms extended toward the sticks).

During this time, children were coded as to whether they helped the experimenter retrieve his or her items (coded “yes” if the child helped at any point during the 40-s period, and “no” if the child did not help even 10 s after the last cue was provided), as well as their latency to respond. For latency, children were given a score of 1–5 corresponding to which cue elicited the child’s help (a score of 5 indicated children did not help after the 4th cue).

#### Comforting Task

A non-costly comforting task was adapted from Svetlova et al. (2010). The experimenter retrieved two toys, noted that those toys are his/her favorite (“These toys are my favorite, they make me happy”) and placed one near the child and out of the experimenter’s reach. The experimenter then proceeded to play with the second toy and pretended to accidentally break it (the toy was configured in such a way that it broke upon handling). The experimenter then expressed 4 cues in successive order in order to elicit the child’s help. Each cue was followed by a 10-s pause in order to allow the child an opportunity to respond. Responding was defined as either providing the second toy to the experimenter or attempting to fix the first toy. During the first cue, the experimenter simply stared at the broken toy and exclaimed “Oh no!” During subsequent cues, the experimenter alternated between looking at the toy and the child and said “I broke my toy!” (second cue), “I am sad, I want another toy” (third cue), and “Can you help me get my other toy?” (fourth cue; with palms extended toward the other toy).

During this time, children were coded as to whether they comforted the experimenter (coded “yes” if the child helped at any point during the 40-s period, and “no” if the child did not act even 10 s after the last cue was provided), as well as their latency to respond. For latency, children were given a score of 1–5 corresponding to which cue elicited the child’s action (a score of 5 indicated the child did not help after the 4th cue).

#### High- and Low-Cost Resource Giving

In these last two tasks, adapted from Chernyak and Kushnir (2013), children were given the opportunity to give to a doll that was sad. These tasks were similar to the comforting task described above but add a personal cost to the action because children had to sacrifice an object that they could keep in order to comfort an agent (see Svetlova et al., 2010 for a similar approach). The adapted task employed a two-step design in which children were first introduced to a doll that was described as feeling “very sad.” In the first step (high-cost giving), the child was then shown a resource (an attractive toy) and told that they could either keep it or give it to the doll to make the doll feel better. The child was then asked whether s/he would like to keep the toy for him/herself or whether s/he would like to give it to the doll to make the doll feel better and provided a box to place the resource into if they wished to give it to the doll. If the child did not respond with an answer, s/he was re-prompted two more times and then provided the resource if no response was given after the last re-prompt. Preliminary analyses revealed that this occurred for only a very small number of children (*n* = 7), who were excluded from any analyses or calculations involving this task. This task was defined as high-cost because the child had only one resource to either keep or give and was done first to prevent the larger variation in resources the children had obtained that occurs in the next step.

During the second step (low-cost giving), children were shown a new doll, told that the new doll was also feeling upset, and then shown three toys, that they could then allocate however they wished. Children were shown two boxes – one for the doll, and one for the child – and prompted to split the three toys into the boxes. If children left any toys unallocated, they were re-prompted until each toy was assigned to either the child or the doll. The number of toys that children gave to each doll during each step was recorded. This task was defined as low-cost because the child had three resources and thus could give something without sacrificing everything. Moreover, this task was completed after the high-cost giving task (thus giving the child an opportunity to keep one item in his or her possession), thus lessening the cost demands on the child.

#### Objective Wealth

Regular income is rare in this region of Zambia and health research typically use an assessment of household wealth. In the initial baseline survey, care-givers were asked if the home has specific assets (a radio, TV, stove, bicycle, farm animals) and access to utilities such as electricity and running water. A composite measure was created based on these responses and standardized (*z*-scored) across the sample.

#### Parent Beliefs About Inequality

We added beliefs about inequality in mothers’ questionnaires in order to assess the impact of *subjective* perceptions of wealth and inequality. The first question (Village Inequality Belief) asked the mother “Which statement best characterizes your village?” with four response options: (1) Everyone has about the same; (2) Some people have a little more than others; (3) Some people have a lot more than others; and (4) A few people have much more than everyone else. The next two questions assessed *subjective wealth* status at both the village (question 2; Local Subjective Wealth) and country level (question 3; Global Subjective Wealth): “Thinking of your village/country, do you think that you have much more or much less than other people in your village/country.” The five response options were: (1) a lot less; (2) a little less; (3) about the same; (4) a little more; and (5) a lot more. Finally, the last question asked the mother: “How much of a problem do you think wealth inequality is in your country?” (Inequality as a Problem Belief). Responses were on a five-point scale: (1) not a problem at all; (2) a small problem; (3) a moderate problem; (4) a big problem; and (5) a very big problem.

#### Child Cognitive Ability

Children’s cognitive abilities were assessed as part of the larger health study using the Bayley Scale for Infant and Toddler Development, Third Edition (BSID-III). The cognitive sub-scale of the BSID-III includes a set of age appropriate tasks that the child is asked to complete, focused on various cognitive skills including object relatedness, pattern recognition, and memory. Standardized scores for the BSID-III are based on norms from a United States sample, and should not be extended to other populations which likely have different normative trajectories of cognitive development (Cromwell et al., 2014). Therefore, children’s raw scores on the cognitive sub-scale were established by summing the number of items successfully completed for each; raw scores were then converted to *z*-scores by standardizing within the study population.

All assessments were administered in the local language that was most familiar to the family and administered in the family’s home by a local researcher conversant in both English and the family’s local dialect (if not English).

## Results

Preliminary analyses showed no gender, age, child height, or treatment effects (effect of intervention), so data were collapsed across these variables. For ease of comparison, we analyze all behaviors categorically (whether children opted into the target behavior or not). However, all reported results remained consistent when looking at prosocial behavior on continuous metrics (i.e., latency to help, rather than whether the child helped). See Supplementary Analyses for details.

**FIGURE 1:**
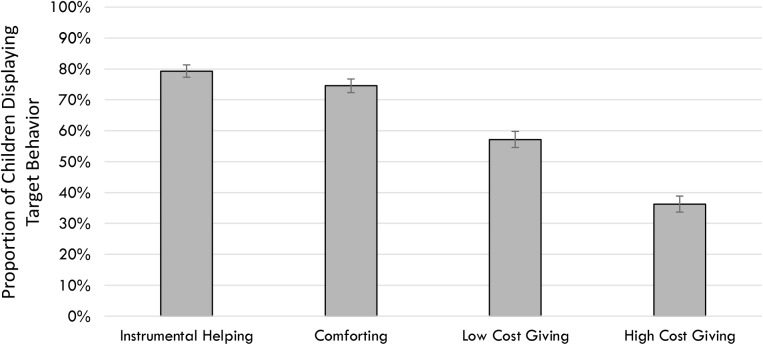
Proportion of children displaying each target behavior. Bars represent ±1 standard error.

We first investigated the *rates* of prosocial behavior across the three tasks (Figure 1). Children displayed instrumental helping and comforting at very high rates (approximately 75–80%), similar to rates found in Western samples investigating this age range (Svetlova et al., 2010). In contrast with prior work using Western samples (Chernyak and Kushnir, 2013), however, rates of high- and low-cost resource giving were markedly lower, though we note that the age-group sampled here was on average, younger, and thus direct comparisons are not possible.

Importantly for the hierarchy of costs model, the *relative rates* of each of the behaviors were consistent with what is reported in Western samples: instrumental helping most common, followed by comforting, and followed by low-cost and then high-cost resource giving (see Svetlova et al., 2010).

Within-subjects McNemar’s tests comparing the rates of each behavior to one another showed that the rate of each target behavior was significantly different from the others (all *p*s < 0.001, with the exception of the comparison between instrumental helping and comforting: *p* = 0.057).

Spearman’s rho correlations (see Table 1) showed that all behaviors, with the exception of high cost resource giving were significantly related to one another. In contrast, high-cost resource giving was only related to low-cost resource giving, suggesting a dissociation between lower-cost behaviors such as helping and comforting and higher-cost behaviors such as giving away one’s only resource.

**Table 1:** Spearman’s rho correlations for each of the target behaviors.

	Instrumental helping	Comforting	Low cost resource giving	High cost resource giving
Instrumental helping	–	**0.408^∗∗∗^**	**0.213^∗∗∗^**	0.069
Comforting		–	0.140^∗∗^	0.057
Low cost resource giving			-	**0.317^∗∗∗^**
High cost resource giving				-


*^∗^p < .*05*; ^∗∗^p < .*01*; ^∗∗∗^p < .*001*. Significant effects are displayed in bold.*

We next examined the questionnaire measures of wealth and inequality. Table 2 shows descriptives and correlations among the key variables of interest: Objective Wealth (*z*-score) and the four questionnaire items (Village Inequality Belief, Local Subjective Wealth, Global Subjective Wealth, and Inequality as a Problem Belief). Generally, people reported that their village was characterized by inequality. Caregivers reported, on average, a 3.42 on a scale of 1–4, very close to the statement that some people have a lot more than everyone else. The majority of the sample also reported themselves as being poorer relative to others in their village and their country (one sampled *t*-tests comparing responses to the midpoint of 3, “about the same,” *t*s < -20.0, *p*s < 0.001). Finally, people reported on average that inequality was at least a moderate to big problem (reporting an average of 3.45 on a scale of 1 to 5, where 1 indicated that inequality was not a problem and 5 indicated that it was a very big problem).

**Table 2:** Pearson’s correlations for each of the inequality/subjective wealth items and objective wealth the less likely they were to believe that inequality was a big problem in Zambia.

	Objective wealth	Village inequality belief	Local subjective wealth	Global subjective wealth	Inequality as a problem belief
Objective wealth	–	0.063	**0**.200***	**0**.167**	-0.053
Village inequality belief (1–4)		–	**-0**.166**	**-0**.129*	**0**.102*
(*M* = 3.42; *SE* = 0.046)					
Local subjective wealth (1–5)			-	**0.548^∗∗∗^**	**-0**.148**
(*M* = 2.07; *SE* = 0.044)					
Global subjective wealth (1–5)				-	**-0**.154**
(*M* = 1.93; *SE* = 0.044)					
Inequality as a problem belief (1–5)					-
(*M* = 3.45; *SE =* 0.067)					


*^∗^ p < .05; ^∗∗^ p < .01; ^∗∗∗^ p < .001. Significant effects are displayed in bold.*

As shown in Table 2, objective wealth was correlated with both local and global subjective wealth, but not correlated with belief that inequality is a problem. Both local and global subjective wealth were very strongly correlated, and moderately negatively correlated with the belief that inequality is a problem. Thus, the richer people perceived themselves to be, the less likely they were to believe inequality was a problem.

For the final set of analyses, we examined whether objective wealth, subjective wealth and inequality beliefs predicted each of the target prosocial behaviors. For these analyses, we ran binary logistic regressions using each of the target behaviors as the dependent variable, and Objective Wealth (*z*-score), and answers to each of the four inequality/subjective wealth questions as predictors. We also initially checked for any effects of Age, Child’s Sex, Intervention Group, Child Height (as a proxy for physical development), and General Cognitive Ability (taken from the cognitive subscale of the BSID-III; *z*-scored) and removed these if they were non-significant. Unless otherwise noted, these were not significant.

**FIGURE 2:**
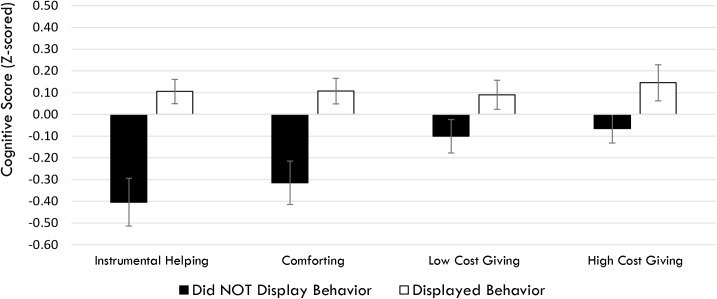
*Z*-scores (bars reflect standard error) for the cognitive scores across each of the prosocial behaviors.

For Instrumental Helping, there was a significant effect of Cognitive Ability (*B* = 0.524, *SE*(*B*) = 0.132, *p* < 0.001), and a significant effect of Local Subjective Wealth (*B* = 0.458, *SE*(*B*) = 0.211, *p* = 0.030). Children with higher cognitive ability and children with mothers who indicated having more than others in their village were more likely to engage in instrumental helping. No other effects reached significance (all *p*s > 0.15). For Comforting, there was a significant effect of Cognitive Ability, *B* = 0.421, *SE*(*B*) = 0.122, *p* = 0.001, and no other significant effects (all *p*s > 0.15). Thus, children with higher cognitive ability were also more likely to engage in comforting behaviors (Figure 2).

For Low Cost Giving, there was a significant effect of Village Inequality Belief, with children whose mothers believed the village was characterized by larger inequality having children who were more likely to share at least one toy out of three with the doll, *B* = 0.331, *SE*(*B*) = 0.121, *p* = 0.006, and no other significant effects (all *p*s > 0.25). For High Cost Giving, there was a significant effect of Cognitive Ability, *B* = 0.269, *SE*(*B*) = 0.123, *p* = 0.029, and no other significant effects (all *p*s > 0.15) (Figure 2).

Finally, given that high- and low-cost giving both took place in the context of pretend play (giving resources to a doll, as opposed to an experimenter), we explored the possibility that rates of giving were lower than what is observed in Western samples because children in Zambia were less familiarized with such pretend scenarios. Although we did not assess pretend play directly, the larger public health study did administer a question on whether the parent had ever read books or looked at picture books with the child. Indeed, less than half (40.6%) of mothers answered affirmatively to that question. Adding this question into the model predicting low-cost giving showed a significant relation between opting into low-cost giving and whether the parent read books to the child, *B* = 0.478, *SE*(*B*) = 0.219, *p* = 0.029, but not high-cost giving. Thus, one mechanism that influences children’s prosocial behavior toward toys and puppets may be the extent to which they are exposed to pretend scenarios more generally.

## Discussion

Despite a large body of recent research on the development of prosocial behaviors, no studies have examined all three components of prosociality outside of wealthy Western countries. We thus began this work by investigating the universality and cultural variability of two recent claims: that by the 3rd year of life, young children generally show high rates of instrumental helping and comforting with others (Warneken and Tomasello, 2006; Svetlova et al., 2010); and second, that, prosocial behavior follows a hierarchy of costs model (Warneken and Tomasello, 2009) with helping appearing first, followed by comforting, and then followed by costly resource giving (Svetlova et al., 2010; Brownell, 2012). We found support for both of these claims in a sample that was vastly different than those studied in prior work, characterized by high inequality, and in children with relatively little exposure to modern schooling. Children tended to show high rates of prosociality at the same point in development previously laid out in prior work. We note that these first set of analyses was aimed to provide a descriptive of the rates of prosociality within this society. Thus, unlike prior work off of which our study was based (Warneken and Tomasello, 2009; Svetlova et al., 2010; Chernyak and Kushnir, 2013), we did not include control conditions that were included in this prior work.

Our results, combined with this prior work, suggest that children take a cost-based approach to prosocial behavior (Sommerville et al., 2018): rates of prosocial behavior varied hierarchically based on the cost of the behavior (with instrumental helping, which is generally considered the lowest cost appearing first; followed by comforting, which involved expending greater effort to soothe the experimenter; followed by giving up their own resources in order to alleviate the distress of others). The fact that prosociality appears governed by cost even in a very poor sample suggests that this may be a cognitive universal.

We also find correlations among the lower cost behaviors, though not the higher cost behaviors. We note that this finding is slightly divergent from prior work, which has found little to no correlations (Dunfield and Kuhlmeier, 2013; Dunfield, 2014) among varieties of prosocial behavior. One possibility is the difference in samples: it is possible that prosocial behavior is viewed as a unitary construct among rural Zambian children, but not among Western children that may be frequently exposed to some behaviors through schooling (e.g., sharing), others through household chores (e.g., instrumental helping) (Hammond and Carpendale, 2014), and yet others through mental state talk (e.g., comforting; Drummond et al., 2014). The tasks used here also may have been more similar in eliciting empathy than in prior work, given that the experimenter or the doll were presented as sad.

Child cognitive ability was related to nearly every form of prosocial behavior suggesting that general cognitive development plays a role in children’s abilities to display prosociality. This particular finding underscores the importance of going beyond studying age-related changes and studying the cognitive predictors and underpinnings of why those age-related changes occur. Recent work has taken an interest in understanding the cognitive underpinnings of prosocial behavior (Blake et al., 2015b; Cowell et al., 2017; Steinbeis and Over, 2017; Chernyak et al., 2018). Our study adds to this work by highlighting that general cognitive ability explains a substantial portion of the variance in prosocial behavior, underscoring the importance that various domain-general abilities may serve as important pre-requisites for our prosocial tendencies. We encourage future work to continue to focus on how cognition underlies our behavioral capacities in the prosocial domain.

We find that rates of opting into low- and high-cost giving were markedly lower than those observed in prior work using a similar paradigm (Chernyak and Kushnir, 2013; Chernyak et al., 2017). Prior work does find variability in costly resource sharing across cultures (House et al., 2013; Blake et al., 2016), though the majority of this work, to our knowledge, has investigated older age groups. Though we did not include a Western sample as a direct comparison, one possibility, of course, is that our sample was slightly younger than that observed in past work, and thus costly sharing was too difficult for this age group. Another, and more intriguing possibility, is that the global culture in which children were raised made the toys too high-value to give away for the sake of someone else’s comfort. Thus, as noted above, children’s behavior may have been governed by the cost of the action.

Another possibility is that because the high- and low-cost resource sharing task took place in the context of pretend play, only children who were familiarized with such symbolic play opted into resource giving toward a toy doll. Prior work does find marked cultural differences in symbolic play (Callaghan et al., 2011). Moreover, prior work shows relations between parents’ use of emotion talk during book reading and children’s prosocial behavior (Brownell et al., 2013). Pretend play, whether in the context of storybooks or symbolic objects, may thus provide one method through which children are exposed to and have opportunities to consider the emotional and physical needs of others. Our exploratory analyses showed a strong correlation between children’s exposure to storybooks and their resource sharing, which held even when considering the effect of other confounding variables (i.e., social class and perceived access to resources). If this is the case, then our work points to the importance of considering ecological cultural validity (e.g., the extent to which toys and animal puppets, or resources in general, are valued by the sampled culture) in conducting cross-cultural investigations. Future work should investigate these possibilities more directly.

Our wealth and inequality questionnaires allowed us to explore the extent to which environmental variables shape our prosocial behavior, thus pointing to a source of individual differences in early prosociality. Prior work has found that social class is related to altruistic giving, although the direction of effects varies across studies. Our results find that family wealth did not relate to children’s prosocial behavior, once we controlled for other correlates of objective wealth, namely, subjective wealth and beliefs that inequality was a problem. However, we note that our measures of household assets differ markedly from measures used in prior work, and also that given the high degree of poverty in the sample population, there was not a large range of objective wealth.

The extent to which mothers believed that inequality was a problem predicted children’s rates of opting into low-cost resource sharing, pointing to the potential of parent-child transmission to prosocial behavior. Though this is a speculative possibility, one mechanism may be that mothers who discuss, emphasize, and elaborate on issues of inequality may also have children who are more willing to expend resources to comfort someone in distress. Such a possibility would be consistent with work generally finding that parent-child discussion of others’ mental states predicts empathetic helping in toddlers (e.g., Drummond et al., 2014). Future work should directly include measures of parents’ discussion of inequality in order to more directly study how parent-child conversation surrounding inequality shapes children’s own beliefs, and subsequently, their prosocial behavior.

Finally, we found that subjective local wealth predicted instrumental helping, even when controlling for other class variables, suggesting that children of mothers who perceived themselves as having more than others around them were also more willing to help others attain their goals. One possibility is that these children were more familiarized with instrumental helping and reciprocal exchanges (Cortes Barragan and Dweck, 2014), since mothers may have felt more obligation to help others more generally.

In summary, this investigation points to the value of including understudied populations in developmental work, both as a way to validate existing theories on prosociality and as a way to explore potential individual differences more generally. In general, we replicate past work, and also join recent efforts in exploring the impact of culture on the diversity of prosocial behavior. In studying how previously documented effects do and don’t hold across cultures, we hope to impart the need for broader, and more representative samples within developmental psychology.

## Ethics Statement

This study was carried out in accordance with the recommendations of Boston University and the ethics board at ERES Converge in Zambia (protocol number 2013Dec010) with written informed consent from all participants or guardians (for child participants). All subjects gave written informed consent in accordance with the Declaration of Helsinki. The protocol was approved by the Boston University and the ERES Converge in Zambia (protocol number 2013Dec010). Parental consent was obtained for child participants.

## Author Contributions

All authors designed the studies and questionnaires. PR led data collection in Zambia. NC conducted data analyses and wrote the first draft of the manuscript. PB provided critical revisions to the manuscript. All authors provided revisions and suggestions to the manuscript.

## Conflict of Interest Statement

The authors declare that the research was conducted in the absence of any commercial or financial relationships that could be construed as a potential conflict of interest.

## Footnotes

1 Gini coefficient is a measure of income inequality within a region, with 0 representing perfect equality, and 1 representing a society in which one individual owns all of the income/wealth.
