# Supplementary material for: Varieties of Young Children’s Prosocial Behavior in Zambia: The Role of Cognitive Ability, Wealth, and Inequality Beliefs
Source: Front Psychol. 2018 Nov 16;9:2209. doi: 10.3389/fpsyg.2018.02209 (PMC6250828; doi:10.3389/fpsyg.2018.02209)
Supplement: Supplementary file 1 [file Data_Sheet_1.docx]

**Supplementary Analyses**

Here, we analyzed predictors of prosocial behaviors when prosocial behaviors were assessed continuously (rather than categorically).

**Instrumental Helping**

For instrumental helping, children were given a score of 1-5 corresponding to the cue after which children helped (see Methods). Mean latency to help was after 2.544 cues (*SE* = 0.083). Thus, higher scores indicated that children were slower to help. Consistent with what is reported in the main manuscript, there was a significant effect of Cognitive Ability, *B* = -0.199, *SE(B*) = 0.084, *p* = 0.018, a significant effect of Local Subjective Wealth, *B* = -0.303, *B* = 0.117, *p* = 0.010, and no other significant effects.

**Comforting**

For comforting, children were also given a score of 1-5 corresponding to the cue after which children comforted (see Methods). Mean latency to comfort was after 2.714 cues (*SE* = 0.085). A paired samples t-test showed that children were generally slightly quicker to help than to cofort, *t*(376) = 2.122, *p* = 0.034. Thus, higher scores indicated that children were slower to comfort. There was a marginal effect of Cognitive Ability, *B* = -0.144, *SE*(*B*) = 0.087, *p* = 0.098, a marginal effect of Objective Wealth, *B* = -0.163, *SE*(*B*) = 0.087, *p* = 0.061, and no other significant effects (*p*’s > 0.13). This was consistent with what was reported in the main manuscript, though we note that the effect of Cognitive Ability was only marginal.

**Low Cost Resource Sharing**

For low cost resource giving, children were given a score of 0-3 corresponding to the number of toys given away to the puppet. Children shared an average of 0.825 toys (*SE*  = 0.045). Consistent with what is reported in the main manuscript, there was a significant effect of Village Inequality Belief, *B* = 0.165, *SE*(*B*) = 0.051, *p* = 0.001, a significant effect of whether children were read books by an adult, *B* = 0.321, *SE*(*B*) = 0.090, *p* < .001, and no other significant effects.

**High Cost Resource Giving**

We note that there was no chance to analyze high cost resource giving continuously, since children either did or did not give away their only resource.
